# Documented inpatient aspirin use and in-hospital mortality in non-HIV tuberculous meningitis: a propensity score-weighted retrospective cohort study

**DOI:** 10.3389/fmed.2026.1908481

**Published:** 2026-08-20

**Authors:** Jianbo Chen, Qiong Wu, Xiangzhi Xiao, Sufen Chen, Jue Hu, Zhen Wang, Wengao Zeng

**Affiliations:** 1 Department of Internal Medicine, The Second Hospital of Changsha (West Branch of Changsha Hospital for Maternal & Child Health Care), Changsha, Hunan, China; 2 Department of Neurosurgery, The Affiliated Changsha Central Hospital, Hengyang Medical School, University of South China, Changsha, Hunan, China; 3 Department of Neurology, The Second Hospital of Changsha (West Branch of Changsha Hospital for Maternal & Child Health Care), Changsha, Hunan, China; 4 Department of Neurology, The Affiliated Changsha Central Hospital, Hengyang Medical School, University of South China, Changsha, Hunan, China

**Keywords:** aspirin, in-hospital mortality, inverse probability of treatment weighting, non-HIV, propensity score, tuberculous meningitis

## Abstract

**Background:**

Aspirin has antiplatelet and anti-inflammatory effects that may be relevant to cerebrovascular complications of tuberculous meningitis (TBM), but its association with survival in routine clinical practice remains uncertain. We examined the association between documented inpatient aspirin use and all-cause in-hospital mortality in a large non-HIV TBM cohort.

**Methods:**

We conducted a single-center retrospective cohort study of 1,574 non-HIV patients hospitalized with TBM from October 2013 through January 2024. Exposure was defined as any documented aspirin administration during the index hospitalization. The primary outcome was all-cause in-hospital death. Missing covariates were handled using multiple imputation by chained equations (20 datasets, 20 iterations). Stabilized inverse probability of treatment weights based on 25 prespecified covariates standardized the comparison to the overall cohort. Sensitivity analyses included 1st-99th percentile weight truncation, five-fold cross-fitted augmented inverse probability weighting (AIPW), 1:1 propensity-score matching, and restriction to adults.

**Results:**

Among 1,574 patients, 844 (53.6%) had documented aspirin use and 187 (11.9%) died in hospital. Mortality occurred in 107/844 patients with documented aspirin use (12.7%) and 80/730 patients without documented use (11.0%). The maximum absolute standardized mean difference decreased from 0.202 before weighting to 0.020 after weighting, and the effective sample size remained approximately 1,519. The primary weighted analysis yielded a risk difference of 0.79 percentage points (95% CI,−2.49–4.06), a risk ratio of 1.07 (95% CI, 0.81–1.41), and an odds ratio of 1.08 (95% CI, 0.79–1.48). All 95% confidence intervals crossed the null, and sensitivity analyses produced similar estimates.

**Conclusions:**

Documented inpatient aspirin use was not associated with lower in-hospital mortality after adjustment for measured covariates. Interpretation is limited by unavailable treatment timing and severity measures and by the absence of functional and post-discharge outcomes. These findings represent observational associations.

## Introduction

Tuberculous meningitis (TBM) is the most severe form of tuberculosis and remains associated with substantial early mortality and long-term neurological disability despite effective antituberculosis therapy and adjunctive corticosteroids (1–4). Cerebrovascular complications are common: a systematic review estimated that stroke occurs in approximately 30% of patients with TBM (5). Basal inflammatory exudates, perforator vasculitis, thrombosis, and impaired cerebral perfusion provide a biological rationale for adjunctive antiplatelet therapy (5, 6).

Aspirin may be relevant to TBM through antiplatelet, anti-inflammatory, and pro-resolution effects. Randomized evidence has nevertheless been heterogeneous. A 2010 open-label trial of aspirin 150 mg reported lower 3-month mortality despite a nonsignificant reduction in stroke (7), whereas the childhood trial and other early studies did not establish a consistent survival benefit (8, 9). In HIV-uninfected adults, a 2018 phase 2 trial of 81 mg or 1,000 mg aspirin for 60 days suggested a possible reduction in the composite of new cerebral infarction or death, particularly in microbiologically confirmed TBM, but was not powered for mortality alone (10). More recently, the multicenter ACT-TBM trial found no statistically significant benefit of aspirin or clopidogrel for clinical stroke, radiological infarction, or mortality (11). Meta-analyses have similarly found no clear mortality benefit, although a possible reduction in incident stroke remains supported by low-certainty evidence (12, 13). Current guidance therefore does not recommend routine aspirin for all patients with TBM (2).

To complement trial evidence with routine-care data, we examined the association between documented inpatient aspirin use and all-cause in-hospital mortality in a large cohort of non-HIV patients with TBM. Multiple imputation, inverse probability of treatment weighting (IPTW), augmented inverse probability weighting, propensity-score matching, and prespecified sensitivity analyses were used to adjust for measured confounding and assess the robustness of the findings.

## Materials and methods

### Study design and population

We conducted a single-center retrospective observational cohort study at Changsha Central Hospital, China. Patients diagnosed with TBM between October 1, 2013, and January 1, 2024, were identified from the electronic medical record system. The source cohort has also supported a separate analysis of drug-induced liver injury; the present study addresses a distinct exposure, outcome, and prespecified analysis plan. Eligible patients met accepted clinical research criteria for definite, probable, or possible TBM, integrating clinical manifestations, cerebrospinal fluid (CSF) findings, neuroimaging, and microbiological evidence (14).

**Figure 1:**
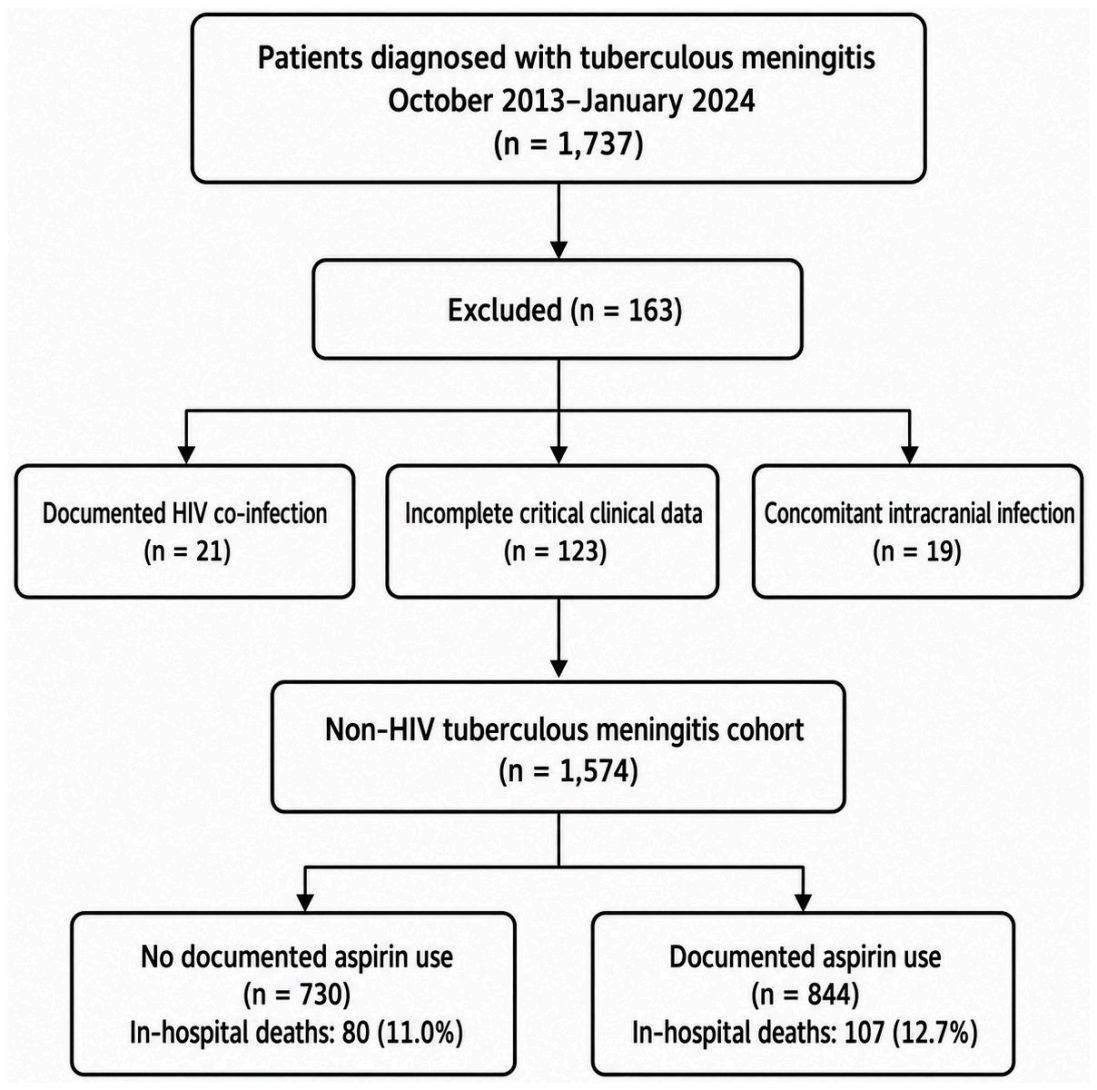
Flow diagram of cohort selection and observed in-hospital mortality according to documented aspirin use.

Exclusion criteria were documented HIV infection, concomitant bacterial or fungal intracranial infection, or incomplete critical clinical data that precluded reliable confirmation of cohort eligibility. Of 1,737 potentially eligible patients, 163 were excluded (21 with HIV infection, 123 with incomplete critical data, and 19 with another intracranial infection), leaving 1,574 non-HIV patients for analysis (Figure 1). The 123 excluded records lacked sufficient core information to determine cohort eligibility and were therefore not candidates for covariate imputation. The index date was the date of the first qualifying TBM hospitalization, and follow-up ended at discharge or in-hospital death.

The study was reported according to the Strengthening the Reporting of Observational Studies in Epidemiology (STROBE) statement and the RECORD-PE extension for pharmacoepidemiological studies using routinely collected health data (15, 30). The Ethics Committee of Changsha Central Hospital approved the study (Approval No. 20250122) and waived written informed consent because the study was retrospective and used anonymized clinical data.

### Data collection and variable definitions

Demographic characteristics, comorbidities, routine blood tests, coagulation indices, cerebrospinal fluid (CSF) measurements, microbiological findings, pulmonary tuberculosis phenotypes, and neuroimaging findings were extracted from the electronic medical records. Continuous variables were defined using values recorded at admission or the earliest available measurement during the index assessment period, consistent with the source-cohort data-collection framework. The binary cerebral-infarction variable indicated whether infarction was documented during hospitalization.

Pulmonary tuberculosis phenotype was categorized as no pulmonary tuberculosis, hematogenous disseminated pulmonary tuberculosis, primary pulmonary tuberculosis, secondary pulmonary tuberculosis, or tuberculous pleuritis. TBM diagnostic certainty was categorized as definite, probable, or possible according to the uniform research case definition (14).

Neuroimaging findings were categorized according to the predominant TBM-related radiological pattern as no TBM-related abnormality, parenchymal type, meningeal type, complication type, or spinal involvement (2, 16). Brain MRI examinations were performed using 1.5-T or 3.0-T systems. Standard imaging protocols included T1-weighted, T2-weighted, fluid-attenuated inversion recovery, and contrast-enhanced T1-weighted sequences, with diffusion-weighted, apparent diffusion coefficient, and susceptibility-sensitive sequences obtained when clinically indicated.

No TBM-related abnormality was defined as the absence of meningeal enhancement, focal parenchymal lesions, hydrocephalus, infarction, or other imaging abnormalities attributable to TBM. Parenchymal-type disease included cerebral infarction, tuberculoma, cerebritis, or other focal or diffuse parenchymal abnormalities. Meningeal-type disease was characterized predominantly by linear or nodular leptomeningeal enhancement, particularly involving the basal cisterns, sylvian fissures, or tentorium. Complication-type disease included hydrocephalus, cerebral edema, hemorrhage, or multiple infarctions. Spinal involvement included spinal leptomeningeal enhancement, arachnoiditis, subarachnoid adhesions, or spinal cord signal abnormalities attributable to tuberculosis.

A composite microbiological-evidence variable was defined as positive when at least one of the following tests was positive: CSF mycobacterial culture, CSF acid-fast bacilli smear, CSF Mycobacterium tuberculosis DNA testing, or a mycobacterial drug-resistance gene assay (14, 16, 17). Hyponatremia was defined as a serum sodium concentration below 135 mmol/L (18).

Non-HIV secondary immunodeficiency was defined as documented acquired impairment of immune function present before or at the index assessment. This included clinically relevant lymphocyte depletion, such as a CD4+ T-cell count below 200 cells/μl or another documented lymphocyte-subset abnormality, or pre-existing immunosuppressive treatment with agents such as systemic glucocorticoids, calcineurin inhibitors, cytotoxic drugs, or biological immunosuppressants (19). Corticosteroids initiated as adjunctive treatment for TBM during the index hospitalization were not considered evidence of pre-existing immunodeficiency.

### Exposure

The exposure was any documented aspirin administration during the index TBM hospitalization. Patients without documented administration formed the comparison group.

### Outcomes

The primary outcome was all-cause in-hospital mortality during the index TBM admission. Cerebral infarction recorded during hospitalization was summarized descriptively. Modified Rankin Scale scores, bleeding outcomes, and post-discharge outcomes were unavailable.

### Covariates

The propensity-score model included 25 variables selected before outcome modeling on the basis of clinical plausibility, prior TBM literature, and availability in the source dataset: age, sex, C-reactive protein, D-dimer, neutrophil count, hepatitis B, non-HIV immunodeficiency, prothrombin time, intracranial pressure, TBM diagnostic certainty, international normalized ratio, albumin, diabetes mellitus, pulmonary tuberculosis phenotype, creatinine, CSF chloride, CSF white-cell count, CSF glucose, CSF protein, platelet count, hemoglobin, procalcitonin, hyponatremia, drug-resistant tuberculosis, and composite microbiological evidence. Age and intracranial pressure were modeled using natural cubic splines with 3 degrees of freedom. Prespecified right-skewed biomarkers were transformed using the natural logarithm of 1+x to reduce right-skewness (20, 21).

### Missing data

Missingness among modeled covariates was low (maximum approximately 1.2%). Missing covariate values were handled using multiple imputation by chained equations with 20 imputed datasets and 20 iterations (22, 23). Predictive mean matching was used for incomplete continuous variables; categorical variables were imputed according to their measurement level. Aspirin exposure and in-hospital mortality were not imputed. Propensity-score estimation and outcome analysis were performed separately within each imputed dataset (24, 25), and the resulting association estimates were combined using Rubin's rules (24, 25). Convergence diagnostics and logged imputation events were reviewed before modeling.

### Statistical analysis

Continuous variables are summarized as mean (standard deviation) when approximately symmetric and as median (interquartile range) otherwise; categorical variables are summarized as number (percentage). Baseline *P* values were not used to select confounders. Table 1 reports standardized mean differences (SMDs) for the original clinical variables, whereas propensity-score balance was assessed on the transformed model-matrix terms within each imputed dataset. Absolute SMDs below 0.10 were considered acceptable.

**Table 1:** Characteristics at index assessment according to documented aspirin use.

Characteristic	No aspirin (*n* = 730)	Aspirin (*n* = 844)	Absolute SMD	Missing, *n*
Age, years	42.24 ± 21.03	41.87 ± 21.03	0.017	0
C–reactive protein, mg/L	5.00 [5.00, 13.70]	5.00 [5.00, 14.60]	0.007	3
D–dimer, mg/L	0.60 [0.60, 0.60]	0.60 [0.60, 0.60]	0.034	11
Neutrophil count × 10^9^/L	3.50 [2.58, 3.54]	3.50 [2.58, 4.06]	0.049	19
Prothrombin time,s	10.60 [10.60, 11.90]	10.60 [10.60, 11.80]	0.017	19
Intracranial pressure, mmH2O	150.00 [120.00, 190.00]	150.00 [125.00, 200.00]	0.096	0
INR	1.00 [1.00, 1.06]	1.00 [1.00, 1.05]	0.009	18
Albumin, g/L	35.16 ± 5.80	34.83 ± 5.76	0.058	1
Creatinine, μmol/L	48.20 [36.00, 66.00]	47.40 [36.00, 63.45]	0.016	8
CSF chloride, mmol/L	125.00 [116.80, 125.00]	123.00 [114.00, 125.00]	0.109	0
CSF white–cell count, × 106/L	10.00 [10.00, 30.00]	10.00 [10.00, 80.00]	0.047	0
CSF glucose, mmol/L	2.20 [2.20, 3.30]	2.20 [2.20, 3.15]	0.112	0
CSF protein, g/L	0.90 [0.54, 1.17]	0.90 [0.62, 1.60]	0.174	0
Platelet count, × 10^9^/L	217.00 [158.00, 266.00]	217.00 [150.00, 277.00]	0.056	15
Hemoglobin, g/L	115.00 [104.17, 126.00]	114.00 [105.00, 126.00]	0.013	0
Procalcitonin, ng/mL	0.04 [0.03, 0.09]	0.04 [0.03, 0.09]	0.070	3
Sex: Male	470 (64.4%)	565 (66.9%)	0.054	0
Female	260 (35.6%)	279 (33.1%)	0.054	
Hepatitis B: No	670 (91.8%)	767 (90.9%)	0.032	0
Yes	60 (8.2%)	77 (9.1%)	0.032	
Immunodeficiency: No	506 (69.3%)	616 (73.0%)	0.081	0
Yes	224 (30.7%)	228 (27.0%)	0.081	
TBM diagnostic certainty: Definite	250 (34.2%)	303 (35.9%)	0.035	0
Probable	451 (61.8%)	502 (59.5%)	0.047	
Possible	29 (4.0%)	39 (4.6%)	0.032	
Diabetes mellitus: No	686 (94.0%)	805 (95.4%)	0.063	0
Yes	44 (6.0%)	39 (4.6%)	0.063	
Pulmonary TB phenotype: No pulmonary TB	156 (21.4%)	191 (22.6%)	0.030	0
Hematogenous disseminated TB	220 (30.1%)	270 (32.0%)	0.040	
Primary pulmonary TB	23 (3.2%)	30 (3.6%)	0.022	
Secondary pulmonary TB	329 (45.1%)	350 (41.5%)	0.073	
Tuberculous pleuritis	2 (0.3%)	3 (0.4%)	0.015	
Hyponatremia: No	541 (74.1%)	591 (70.0%)	0.091	0
Yes	189 (25.9%)	253 (30.0%)	0.091	
Drug–resistant TB: No	688 (94.2%)	788 (93.4%)	0.037	0
Yes	42 (5.8%)	56 (6.6%)	0.037	
Any microbiological evidence: No	667 (91.4%)	781 (92.5%)	0.043	0
Yes	63 (8.6%)	63 (7.5%)	0.043	

Data are mean ± SD, median [interquartile range], or *n* (%). For binary variables, the displayed level is specified in the characteristic column. This table shows complete–case descriptive summaries on the original clinical scale before multiple imputation. Propensity-score balance diagnostics were calculated on transformed model-matrix terms within each of 20 imputed datasets; the maximum unweighted SMD of 0.202 corresponded to log-transformed CSF protein. SMD, standardized mean difference; TBM, tuberculous meningitis; CSF, cerebrospinal fluid; INR, international normalized ratio.

For the primary analysis, a logistic propensity-score model was fitted separately in each imputed dataset. Stabilized inverse probability of treatment weights were then constructed to standardize the distribution of measured covariates to the overall analyzed cohort (26, 27). Positivity was assessed using propensity-score overlap, score ranges, weight percentiles, maximum weights, and effective sample size. Weighted standardized risks were used to estimate the adjusted risk difference (RD), risk ratio (RR), and odds ratio (OR) for in-hospital death with 95% confidence intervals. Covariate balance was summarized across imputed datasets and displayed using Love plots.

Sensitivity analyses included truncation of stabilized weights at the 1st and 99th percentiles; five-fold cross-fitted AIPW; 1:1 nearest-neighbor propensity-score matching without replacement using a caliper of 0.2 standard deviations of the logit of the propensity score, standardized to patients with documented aspirin use; and restriction to adults aged 18 years or older (28, 29).

For AIPW, treatment and binary-outcome nuisance models were fitted using logistic regression within each training fold; the outcome model included aspirin exposure and the prespecified covariates. Bias-reduced logistic regression was used as a fallback when standard logistic regression failed, and predicted propensity scores were bounded at 0.01 and 0.99. All analyses were performed separately within each imputed dataset and pooled using Rubin's rules. Pooled results were reported only when at least 16 of 20 imputed datasets yielded valid estimates; all analyses reported here met this criterion.

All tests were two-sided. Precision was interpreted from confidence intervals rather than from a binary significance threshold alone. Analyses were conducted in R version 4.5.3 using the mice, WeightIt, MatchIt, cobalt, survey, and brglm2 packages.

## Results

### Cohort characteristics

The final cohort comprised 1,574 non-HIV patients with TBM; 844 (53.6%) had documented aspirin use and 730 (46.4%) did not. Overall, 187 patients (11.9%) died during hospitalization. Mortality was 12.7% (107/844) in the aspirin group and 11.0% (80/730) in the no-aspirin group. Among 1,382 adults aged at least 18 years, 168 died and 737 received aspirin.

The source dataset recorded whether aspirin was administered during hospitalization but did not contain analyzable information on initiation date, dose, treatment duration, indication, adherence, or discontinuation.

Selected index-assessment characteristics are shown in Table 1. The largest original-scale differences involved CSF measures: patients with documented aspirin use had higher CSF protein, a broader distribution of CSF white-cell counts, and lower CSF chloride. Table 1 presents SMDs for the original clinical variables; the maximum unweighted SMD of 0.202 used in the propensity-score diagnostics arose from the log-transformed CSF protein term in the model matrix.

### Hospital-recorded cerebral infarction

Cerebral-infarction status was available for 1,573 of 1,574 patients. Infarction was recorded in 265/1,573 (16.8%): 136/844 (16.1%) in the documented-aspirin group and 129/729 (17.7%) in the no-aspirin group. In-hospital death occurred in 37/265 patients with recorded infarction (14.0%) and 150/1,308 patients without recorded infarction (11.5%).

### Multiple imputation and propensity-score diagnostics

**Figure 2:**
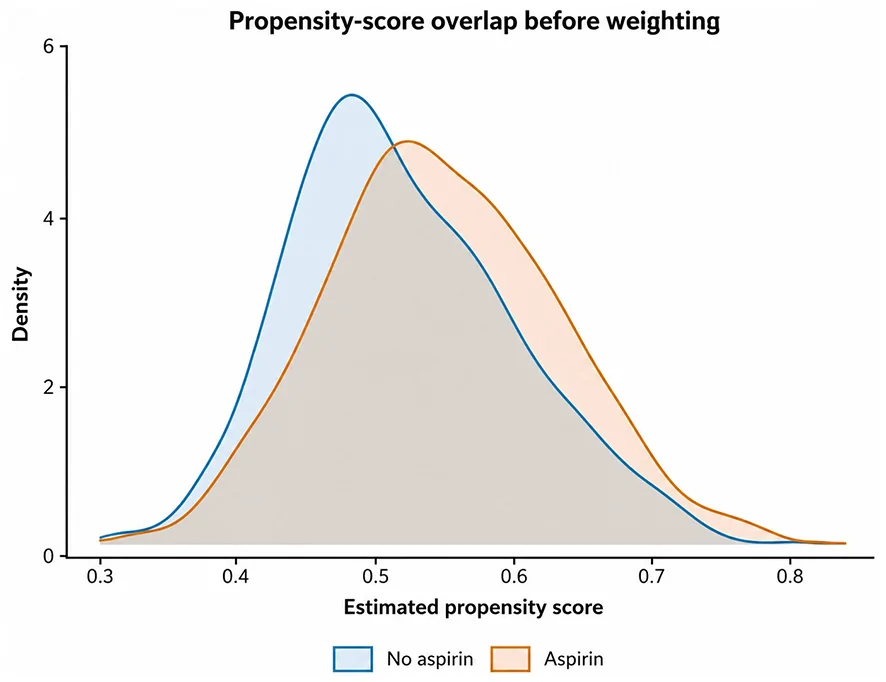
Propensity-score distributions before weighting. Substantial overlap was present between the aspirin and no-aspirin groups.

Twenty imputed datasets were generated successfully, and no logged imputation failures were identified. After imputation, no modeled covariate remained missing. Propensity-score distributions overlapped substantially between groups (Figure 2). Across imputed datasets, propensity scores ranged approximately from 0.276 to 0.865. The 99th percentile of the stabilized weights ranged from 1.57 to 1.63, maximum weights ranged from 3.30 to 3.43, and the effective sample size ranged from 1,518.8 to 1,520.9, indicating no important loss of precision from extreme weighting (Table 3).

**Table 2:** Covariate-adjusted association between documented aspirin use and in-hospital mortality.

Analysis	Target	Risk difference, percentage points (95% CI)	Risk ratio (95% CI)	Odds ratio (95% CI)
Unadjusted	Observed	1.72 (-1.47–4.91)	1.16 (0.88–1.52)	1.18 (0.87–1.61)
IPTW, primary	Overall	0.79 (-2.49–4.06)	1.07 (0.81–1.41)	1.08 (0.79–1.48)
IPTW with 1st-99th percentile weight truncation	Overall	0.88 (-2.36–4.12)	1.08 (0.82–1.42)	1.09 (0.80–1.49)
Cross-fitted AIPW	Overall	2.40 (-5.11–9.91)	1.28 (0.50–3.28)	1.31 (0.47–3.65)
1:1 propensity-score matching	Aspirin	0.42 (-3.32–4.15)	1.04 (0.75–1.43)	1.04 (0.72–1.50)
Adult-only IPTW	Adults	1.54 (-1.97–5.05)	1.14 (0.85–1.53)	1.16 (0.83–1.62)

Positive risk differences and ratios above 1 indicate higher mortality in the documented-aspirin group. Targets are the observed cohort (unadjusted), overall cohort (IPTW/AIPW), aspirin group (matching), and adult cohort (adult-only IPTW). IPTW, inverse probability of treatment weighting; AIPW, augmented inverse probability weighting. Estimates were pooled using Rubin's rules.

**Table 3:** Propensity-score and weighting diagnostics.

Domain	Diagnostic	Result
Participants	No aspirin/aspirin	730/844
Propensity score	Range across imputed datasets	0.276–0.865
Stabilized weight	99th percentile range	1.57–1.63
Stabilized weight	Maximum range	3.30–3.43
Effective sample size	Range across imputed datasets	1,518.8–1,520.9
Absolute SMD	Maximum before IPTW	0.202
Absolute SMD	Maximum after IPTW	0.020
Post–weighting balance	Covariates with absolute SMD ≥0.10	0
Propensity–score matching	Matched sample range	1,258–1,282
Propensity–score matching	Matched per group range	629–641
Propensity–score matching	Maximum post–match absolute SMD	0.084

Ranges summarize the 20 imputed datasets. SMD denotes standardized mean difference; IPTW, inverse probability of treatment weighting.

**Figure 3:**
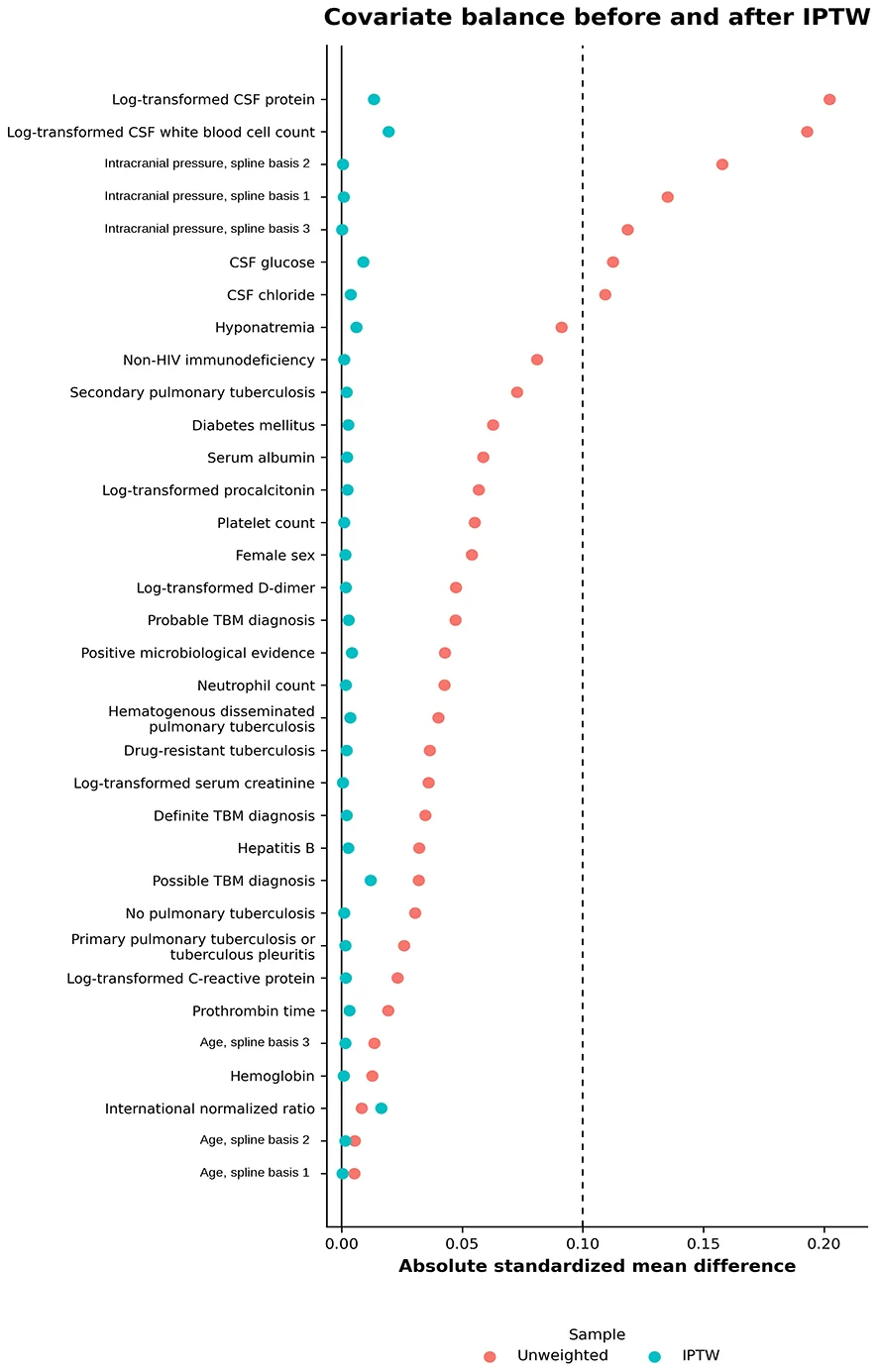
Covariate balance before and after inverse probability of treatment weighting. Points represent mean absolute standardized mean differences across 20 imputed datasets. Age and intracranial pressure were modeled with natural cubic splines (3 degrees of freedom); “spline basis 1–3” denotes model-matrix columns rather than clinical subgroups. The largest unweighted standardized mean difference was 0.202 for log-transformed CSF protein, and the maximum post-weighting value was 0.020.

IPTW achieved excellent measured covariate balance. Across the 20 imputed datasets, the maximum absolute SMD decreased from 0.202 before weighting to 0.020 after weighting, and every weighted model-matrix term had an absolute SMD below 0.10 (Figure 3).

### Primary association with in-hospital mortality

In the unadjusted analysis, documented aspirin use was associated with an absolute mortality difference of 1.72 percentage points (95% CI,−1.47–4.91), an RR of 1.16 (95% CI, 0.88–1.52), and an OR of 1.18 (95% CI, 0.87–1.61).

**Figure 4:**
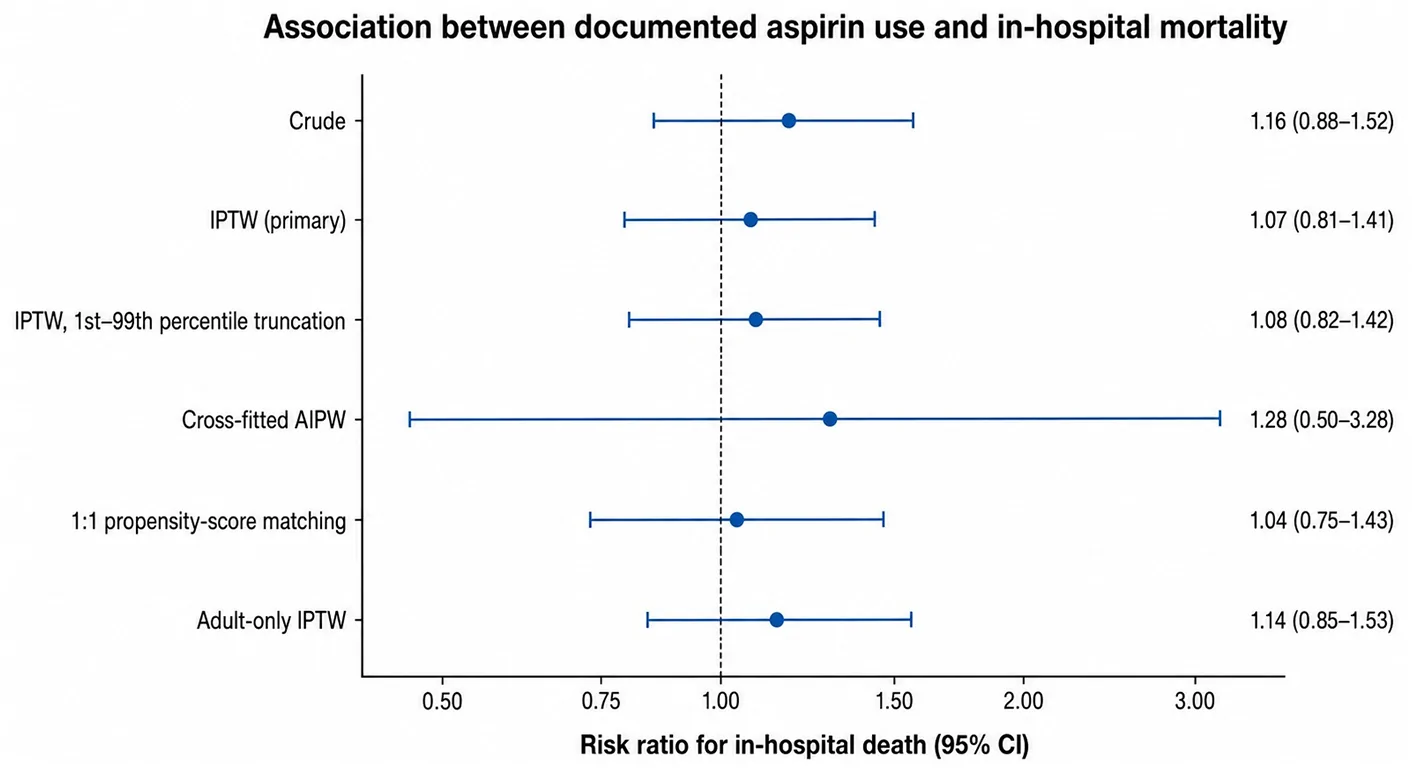
Risk ratios for the association between documented aspirin use and in-hospital mortality across the primary and sensitivity analyses. Points represent pooled estimates and horizontal lines represent 95% confidence intervals. The vertical dashed line indicates no association (risk ratio = 1.00).

In the primary IPTW analysis, the standardized mortality risk was approximately 11.4% without aspirin and 12.2% with aspirin. The pooled RD was 0.79 percentage points (95% CI,−2.49–4.06), the RR was 1.07 (95% CI, 0.81–1.41), and the OR was 1.08 (95% CI, 0.79–1.48) (Table 2 and Figure 4). All 95% confidence intervals crossed the null.

### Sensitivity analyses

Truncating weights at the 1st and 99th percentiles produced nearly identical estimates (RD, 0.88 percentage points; 95% CI,−2.36–4.12; RR, 1.08; 95% CI, 0.82–1.42). The cross-fitted AIPW estimate was less precise, and its 95% confidence interval crossed the null (RD, 2.40 percentage points; 95% CI,−5.11–9.91; RR, 1.28; 95% CI, 0.50–3.28).

Propensity-score matching retained 1,258–1,282 patients across imputed datasets, with 629–641 matched patients in each exposure group. All post-matching SMDs were below 0.10. The matched standardized association was similar to the primary analysis (RD, 0.42 percentage points; 95% CI,−3.32–4.15; RR, 1.04; 95% CI, 0.75–1.43). In adults, IPTW produced an RD of 1.54 percentage points (95% CI,−1.97–5.05) and an RR of 1.14 (95% CI, 0.85–1.53). Across analyses, all 95% confidence intervals included the null value.

## Discussion

In this large single-center cohort of 1,574 non-HIV patients with TBM, documented inpatient aspirin use was not associated with lower all-cause in-hospital mortality after adjustment for measured covariates. The primary estimate was close to the null, although the confidence interval remained compatible with moderate benefit or harm. Weight truncation, AIPW, propensity-score matching, and adult-only restriction produced similar results. The study was not designed as an equivalence or non-inferiority analysis.

Prior randomized evidence has been mixed. The 2010 open-label trial of aspirin 150 mg reported lower 3-month mortality, although stroke reduction was not statistically significant (7). The 2018 phase 2 trial of 81 mg or 1,000 mg aspirin for 60 days suggested a possible reduction in the composite of new infarction or death, particularly among patients with microbiologically confirmed TBM, but was not powered for mortality alone (10). The 2025 ACT-TBM trial found no significant reduction in clinical stroke, radiological infarction, or mortality with aspirin 75 mg or clopidogrel 75 mg (11). Meta-analyses have found no clear mortality reduction while suggesting a possible decrease in incident stroke with low-certainty and sensitivity-dependent evidence (12, 13). Differences in treatment initiation, dose, outcome definition, follow-up, and case mix limit direct comparison with the present routine-care analysis.

Several biases may have influenced the observed association. Clinicians may have prescribed aspirin preferentially to patients with suspected or established vascular complications or evolving severe disease, producing confounding by indication and reverse causation that could bias the aspirin group toward higher mortality. Conversely, classification by any inpatient aspirin use requires survival until treatment is initiated and documented, creating potential immortal-time bias that would tend to favor aspirin (31, 32). Without initiation dates, the direction and magnitude of the combined bias cannot be determined. Heterogeneity in treatment timing, dose, duration, and indication may also have diluted an effect confined to a specific therapeutic strategy or clinical phenotype.

The principal strengths are the large routine-care cohort, transparent handling of limited covariate missingness, assessment of propensity-score overlap, and reporting of measured covariate balance and association estimates on both absolute and relative scales across several analytical strategies.

The study has important limitations. First, aspirin initiation time, dose, duration, indication, adherence, and discontinuation were unavailable, leaving the analysis vulnerable to time-related exposure bias. Second, treatment selection may have reflected evolving clinical status, and key severity measures, including Medical Research Council stage, Glasgow Coma Scale score, intensive-care requirement, mechanical ventilation, and treatment delay, were unavailable; residual confounding by indication is therefore likely. Third, the cerebral-infarction variable lacked onset dates, and modified Rankin Scale, bleeding, and post-discharge outcomes were unavailable. The study consequently could not evaluate incident stroke, disability, safety, or longer-term survival. Finally, the single-center design and exclusion of patients with HIV-associated TBM limit generalizability to other regions, health systems, and HIV-positive populations.

Clinical decisions about aspirin in TBM should continue to be based on randomized evidence, current guidance, and established cardiovascular indications rather than this observational analysis alone. Future studies should align eligibility, treatment assignment, and the start of follow-up; specify treatment initiation within a fixed early window; record dose and duration; measure core TBM severity; and capture incident stroke, hemorrhage, functional status, and post-discharge outcomes.

## Conclusions

Documented inpatient aspirin use was not associated with lower in-hospital mortality after adjustment for measured covariates. Treatment timing, indication, core severity measures, and temporally defined cerebrovascular and functional outcomes were unavailable; causal effects of aspirin initiation therefore could not be estimated. Further studies with predefined treatment windows and clinically relevant outcomes are needed.

## Data Availability

The original contributions presented in the study are included in the article/supplementary material; further inquiries can be directed to the corresponding author.
